# Comparison of Imaging Changes and Pain Responses in Patients with Intra- or Extraosseous Bone Metastases Treated Palliatively with Magnetic Resonance-Guided High-Intensity–Focused Ultrasound

**DOI:** 10.1016/j.jvir.2019.02.019

**Published:** 2019-09

**Authors:** Sharon L. Giles, Matthew R.D. Brown, Ian Rivens, Martin Deppe, Merel Huisman, Young-Sun Kim, Paul Farquhar-Smith, John E. Williams, Gail R. ter Haar, Nandita M. deSouza

**Affiliations:** a Cancer Research UK Cancer Imaging Centre, Magnetic Resonance Imaging Unit, The Royal Marsden Hospital, Sutton, Surrey SM2 5PT, United Kingdom; b Pain Medicine Department, The Royal Marsden Hospital, Sutton, Surrey SM2 5PT, United Kingdom; c Targeted Approaches to Cancer Pain, The Institute of Cancer Research, London, United Kingdom; d Therapeutic Ultrasound, The Institute of Cancer Research, London, United Kingdom; e Philips Radiation Oncology, Vantaa, Finland; f Image Sciences Institute/Imaging Division, University Medical Center Utrecht, The Netherlands; g Department of Radiology and Center for Imaging Science, Samsung Medical Center, Seoul, Korea; h Department of Radiology, Mint Hospital, Seoul, Korea

**Keywords:** MRgHIFU, magnetic resonance-guided high-intensity–focused ultrasound, PRFS, proton resonance frequency shift

## Abstract

**Purpose:**

This study compared changes in imaging and in pain relief between patients with intraosseous, as opposed to extraosseous bone metastases. Both groups were treated palliatively with magnetic resonance-guided high-intensity–focused ultrasound (MRgHIFU).

**Materials and Methods:**

A total of 21 patients were treated prospectively with MRgHIFU at 3 centers. Intraprocedural thermal changes measured using proton resonance frequency shift (PRFS) thermometry and gadolinium-enhanced T1-weighted (Gd-T1W) image appearances after treatment were compared for intra- and extraosseous metastases. Pain scores and use of analgesic therapy documented before and up to 90 days after treatment were used to classify responses and were compared between the intra- and extraosseous groups. Gd-T1W changes were compared between responders and nonresponders in each group.

**Results:**

Thermal dose volumes were significantly larger in the extraosseous group (*P* = 0.039). Tumor diameter did not change after treatment in either group. At day 30, Gd-T1W images showed focal nonenhancement in 7 of 9 patients with intraosseous tumors; in patients with extraosseous tumors, changes were heterogeneous. Cohort reductions in worst-pain scores were seen for both groups, but differences from baseline at days 14, 30, 60, and 90 were only significant for the intraosseous group (*P* = 0.027, *P* = 0.013, *P* = 0.012, and *P* = 0.027, respectively). By day 30, 67% of patients (6 of 9) with intraosseous tumors were classified as responders, and the rate was 33% (4 of 12) for patients with extraosseous tumors. In neither group was pain response indicated by nonenhancement on Gd-T1W.

**Conclusions:**

Intraosseous tumors showed focal nonenhancement by day 30, and patients had better pain response to MRgHIFU than those with extraosseous tumors. In this small cohort, post-treatment imaging was not informative of treatment efficacy.

High-intensity focused ultrasound (HIFU) is emerging as a credible option for palliative treatment of pain from bone metastases 1, 2, a common cause of cancer-related morbidity (3). Studies have evaluated the safety and efficacy of HIFU in radiation-refractory and radiation-naïve populations and shown significant improvements in pain, with a low rate of treatment-related adverse events 4, 5, 6, 7, 8, 9. The mechanism of action may be thermal denervation of the periosteum (2), which exploits cortical bone’s high acoustic absorption and low thermal conduction, so that targeting it results in energy deposition at the periosteal surface (10). This strategy, therefore, may be ineffective for extraosseous metastases, in which the lack of a cortical barrier may mean that ablative energy is transmitted directly into the tumor and misses involved periosteal nerves. Although the cortical integrity of treated tumors has been noted in some previous studies 6, 9, pain response to HIFU in patients with intra- or extraosseous bone metastases remains critically unexplored.

Magnetic resonance-guided HIFU (MRgHIFU) treatments allow accurate depiction of the target and surrounding anatomy. MR imaging-based temperature measurement using a proton resonance frequency shift (PRFS) technique is used to monitor treatments 11, 12, where thermal data are displayed as color overlays superimposed on anatomical images. Raising tissue temperature to ≥56^o^C for only a few seconds is considered ablative (13). After treatment, gadolinium-enhanced T1-weighted (Gd-T1W) imaging can show nonperfused regions indicative of tissue ablation (14). Gd-T1W imaging may also be used to assess outcomes after treatment (6), but the significance of the nonperfused volume (NPV) in relation to the dose delivered or to the treatment response is unclear 8, 15. This study compared imaging changes during and after treatment in patients with intraosseous, as opposed to extraosseous bone metastases, treated palliatively with MRgHIFU. Any changes were related to longitudinal changes in pain scores over 90 days.

## Materials and Methods

### Study Population

This study interrogated an exploratory endpoint in 21 patients with a dominant, painful bone metastasis participating in a prospective, single-arm study (Multicenter Study of Magnetic Resonance-guided High Intensity Focused Ultrasound for Pain Palliation of Bone Metastases [MRgFUS]; NCT01586273) (16). Patients were treated with MRgHIFU at 1 of 3 centers after approval from institutional review boards (REC number: 12/LO/0424, IRB code: 2013-04-050). Patients were provided with a study information sheet before they gave written informed consent for treatment. All patients had a proven diagnosis of bony metastatic disease arising from a primary solid tumor and dominant metastasis pain score of ≥4/10 on a numerical rating scale. Eligibility was determined at screening by using criteria provided in Table 1. Patient and tumor characteristics are provided in Table 2 and were classified as either intraosseous (cortex intact) or extraosseous (cortical breach).

**Table 1:** Eligibility Criteria

Inclusion Criteria	Exclusion Criteria
**Prior to enrollment**	
Adult ≥18 y	Metastasis is from primary bone tumor, lymphoma, myeloma, or leukemia
Patient is capable of communicating and providing informed consent	A communication barrier is present
Weight <140 kg	Patient is enrolled in a conflicting clinical study
Radiologic evidence of bone metastases from any solid tumor	Pain is related to target metastasis due mainly to fracture, impending fracture, or spinal cord compression
Dominant painful bone metastasis (NRS ≥ 4), either refractory to standard of care treatment or standard of care contraindicated or refused by the patient	Target tumor is located in the skull, spine (excluding sacrum), or ribs and sternum (unless exposure to the lung can be avoided)
Patient has been stable taking pain medication for ≥1 wk before proposed HIFU treatment	Surgical stabilization is needed in case of impending fracture (lytic tumor in weight-bearing bone larger than 50% of bone diameter)
Pain is localized to target metastasis or is referred pain arising from it	Pregnancy
Patient has ≤3 painful bone metastases	Prior surgery or minimally invasive treatment of target tumor
Planned HIFU treatment date is ≥4 weeks from the last local treatment of target metastasis	Clinically relevant medical history that could compromise patient safety
**At screening**	
Intended target metastasis is accessible for HIFU	Contraindications to MR imaging or MR contrast medium or sedation
Target tumor diameter is ≤8 cm	Scar along the proposed beam path
Intended target tumor is visible by noncontrast-enhanced MR imaging	Placement of an internal or external fixation device along the proposed beam path or at the target
Distance between the tumor and the skin ≥1 cm	Patient is unable to tolerate required position for treatment
	Target tumor is <3 cm from a critical structure along the proposed beam path or is <1 cm orthogonal to the beam path
	Target is in contact with hollow viscera

Note–Eligibility criteria refer to patients’ suitability for enrolment in the trial before consent for screening investigations. To confirm patients’ suitability for treatment after screening, further eligibility criteria were applied prior to their inclusion.

HIFU = high-intensity focused ultrasound; MR = magnetic resonance; NRS = numerical rating scale.

**Table 2:** Patient and Tumor Characteristics

	Intraosseous Group	Extraosseous Group
Patients		
n (%)	9 (43)	12 (57)
Sex, n (%)	3 Males (33) 6 Females (67)	8 Males (67) 4 Females (33)
Age, y	52.6 ± 9.6	58.1 ± 11.3
Primary tumor site, n (%)		
Breast	6 (67)	2 (17)
Liver	-	4 (33)
Lung	1 (11)	3 (25)
Renal structures	1 (11)	2 (17)
Colorectal area	-	1 (8)
Eccrine glands	1 (11)	-
MRgHIFU treatment site, n (%)		
Pelvis	5 (56)	8 (67)
Ribs	2 (22)	1 (8)
Humerus	1 (11)	1 (8)
Femur	1 (11)	1 (8)
Sacrum	-	1 (8)
Prior EBRT to target metastasis, n (%)	9 (100)	12 (100)
8 Gy 1#	3 (33)	2 (17)
20 Gy 5#	2 (22)	1 (8)
30 Gy 10#	3 (33)	1 (8)
High dose: >30 Gy or multiple treatments	1 (11)	8 (67)
Responder to prior EBRT? CR or PR	3 (33)	6 (50)
Baseline pain from target metastasis, n (%)		
NRS 4–6 moderate pain	3 (33)	5 (42)
NRS 7–10 severe pain	6 (67)	7 (58)

Note–Characteristics of patients (n = 21) treated with MRgHIFU, all of whom had previously received radiation therapy to the target tumor**.** Intraosseous lesions had intact bone cortex along the entire length of the tumor, with no visible periosteal involvement; extraosseous lesions had clear cortical breaches with visible periosteal involvement with tumor.

CR = complete response; EBRT = external beam radiation therapy; MRgHIFU = magnetic resonance-guided high-intensity focused ultrasound; NRS = numerical rating scale; PR = partial response; # = Fractions, ie, the number of treatment episodes over which the total EBRT dose was delivered.

### Baseline Assessments

Pretreatment baseline vital signs and body temperature were recorded, and the target metastasis numerical rating scale pain score was documented in a case report form (CRF). Global pain was assessed using the Brief Pain Inventory short form (BPI-SF) (17). Use of analgesia in the 24 hours prior to treatment was also recorded.

### Treatment Delivery

Treatments were delivered using a Sonalleve HIFU device (Profound Medical, Mississauga, Ontario, Canada) with patients positioned within a 3-T or 1.5-T MR scanner (Philips, Best, The Netherlands). Use of a dampened Aquaflex gel pad (Parker Laboratories Inc., Fairfield, New Jersey) ensured optimal acoustic contact between the patient’s skin and the HIFU device.

After patients were sedated, MRgHIFU treatments were planned on a patient- and tumor-specific basis, using a series of volumetric treatment “cells” of 4-, 8-, or 12-mm diameter (18). For intraosseous tumors, cells were centered on the cortical surface; for extraosseous tumors, additional cells were positioned within the tumor’s soft tissue component.

### Intraprocedural MR Imaging

*T1W* *Imaging*.—Three-dimensional (3D) T1W imaging was acquired over the full extent of the treatment region to confirm that the target metastasis lay within the targeting range of the transducer and to allow treatment planning. If patients moved during treatments, the 3D T1W imaging was reacquired to confirm the patient’s new position and to allow the location of prior sonications to be mapped to the new position.

*PRFS* *Thermometry*.— PRFS thermometry was obtained at 3-second intervals before, during, and after each sonication to evaluate temperature changes in overlying muscle (intraosseous group) and within the tumor (extraosseous group). Sonications were terminated if heating was excessive, or occurred outside the target region, or if patient movement compromised targeting accuracy. After each sonication, PRFS data were reviewed to evaluate the magnitude and extent of thermal changes and to determine the cooling times required to reduce the risk of unwanted heat buildup in surrounding tissues.

*Gd-T1W Imaging.—*On completion of treatments and after administration of 0.2 mL/kg of body weight Gd contrast agent, 3D fat-suppressed Gd-T1W images were obtained. Summary parameters for all sequences are shown in Table E1 (available online on the article’s Supplemental Material page at *www.jvir.org*).

### Assessments after Treatment

After treatment, patients’ pain scores for the treated metastasis were recorded in the CRF. For 30 days after treatment, patients completed a daily diary to record their worst pain score from the treated metastasis and their analgesic consumption. They also completed the BPI-SF at home on days 7 and 14 after treatment and at day 30 when they attended a follow-up appointment to complete the CRF and undergo MR imaging. All investigations were repeated on days 60 and 90 where possible. Investigators reported any adverse events (AEs) that occurred after patients were recruited to the study, in accordance with study and institutional requirements.

### Data Analysis

*Treatment Delivery Parameters.—*The number, diameter, and total volume of treatment cells for each patient were recorded. The duration and power of each sonication was noted; their product provided the applied acoustic energy of each sonication, whose sum for all delivered sonications provided the total acoustic energy for the treatment. Treatment time was measured from first exposure to last.

**Figure 1:**
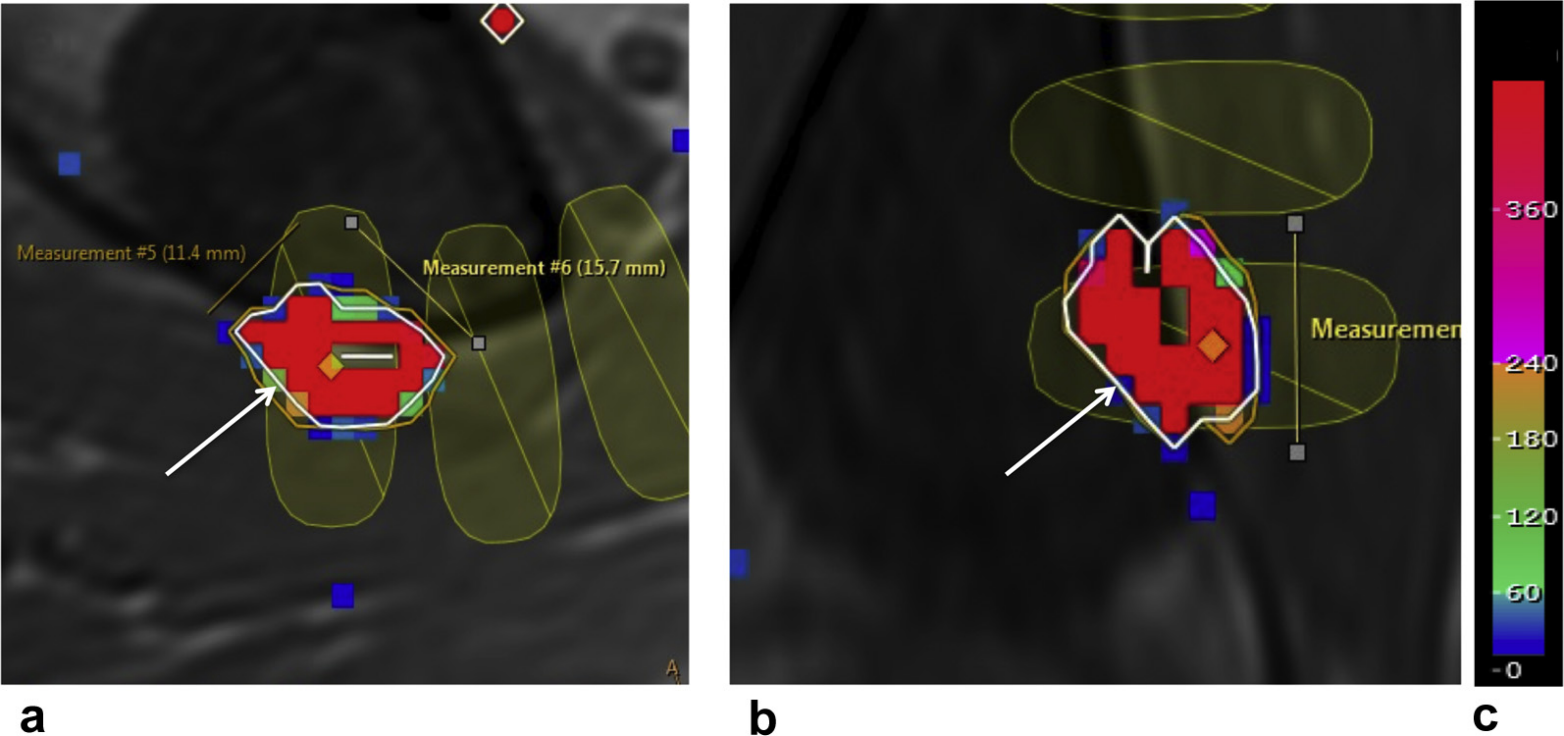
V_240EM_ estimates overlaid on T1W imaging acquired for treatment planning in a patient with an intraosseous tumor. The white arrow pointing at the white outline represents the 240 equivalent minutes (EM) at 43^o^C thermal dose contour in **(a)** the axial and the **(b)** the coronal planes. The colored pixels (the scale given in panel **c**) show the thermal dose in EM within this contour. The product of the 3 largest orthogonal dimensions of the 240EM contour was used to estimate the thermal dose volume (V) of each sonication. The total of these volumes was recorded as the total thermal dose volume, V_240EM_, for each patient. The orange contour represents the 30EM thermal dose contour, and the yellow ellipses show the positions of planned cells.

Thermal changes were measured on PRFS by estimating thermal dose volume, calculated as the product of 3 orthogonal maximum dimensions of the 240 equivalent minutes (EM) at 43^o^C dose contour 12, 19 (Fig 1). The sum of thermal dose volumes for all sonications was the estimate of total thermal dose volume for each patient (V_240EM_). In addition, the maximum temperature recorded in the target region during each sonication was used to calculate the mean maximum temperature (T_M_) from all sonications, for each patient.

*Imaging Changes after Treatment.—*T1W images were used to estimate any changes in tumor diameter from baseline. Gd-T1W images were used to measure NPV by drawing regions of interest on the immediate post-treatment and days’ 30, 60, and 90 images. The total NPV was calculated from the product of totaled regions of interest areas and slice thicknesses. Where no focal NPV was identified, changes were classified as grade 1 change (ill-defined expansion of nonperfused regions) or grade 2 change (definite increase in nonperfusion or reduction in contrast enhancement).

*Treatment Response.—* CRF, diary (local), and BPI-SF (global) pain scores at all post-treatment time points were each compared with baseline (pretreatment) scores. The Pain Severity Index and the Pain Interference Index were also calculated from the BPI-SF (17). A change in analgesic requirement after treatment was assessed from the patient diaries and the CRF.

Treatment response was classified using established criteria (20). Complete response was defined as a BPI-SF worst pain score of zero, without increase in analgesic intake. Partial response was defined as a reduction of ≥2 points in worst pain, without analgesic increase, or an analgesic reduction of ≥25% without increase in worst pain. Pain progression was an increase of ≥2 points in worst pain without analgesic decrease or analgesic increase of ≥25% with worst pain ≤1 point above baseline. No response applied to all other cases. The < or ≥25% change in analgesia was determined by calculating the change in morphine equivalent daily dose (21); for nonopioid medication, where the morphine equivalent daily dose could not be calculated, the magnitude of dose reduction was established through comparison with pretreatment dose. Patients were classified as responders (complete response or partial response) or nonresponders (no response or pain progression) at days 7, 14, 30, 60, and 90 after treatment.

*Adverse Events.—* AEs were classified in accordance with the Clinical Practice Guidelines of the Society of Interventional Radiology (22). AEs were further categorized as definitely, probably, possibly, or unlikely device-related (from MRgHIFU treatment), study-related (from study procedures), or unrelated to treatment.

### Statistical Analyses

Statistical analyses were performed using Prism software (version 7, GraphPad, San Diego, California). The D’Agostino-Pearson test for normality was used to select parametric or nonparametric tests. A *P* value of <0.05 was chosen as the criterion for statistical significance.

Baseline patient and tumor characteristics and treatment delivery parameters for intra- and extraosseous groups were compared using 2-tailed tests for unrelated samples. Where data were normally distributed, unpaired *t*-tests were used; where they were not, Mann-Whitney *U* tests were used. For each group, post-treatment changes in pain scores (CRF, diary, and BPI-SF) were compared using paired *t*-tests and a Bonferroni correction for multiple comparisons.

After treatment, any differences in tumor diameters from baseline were compared using paired *t*-tests for the intraosseous data and a Wilcoxon matched pairs signed rank test for the non-normally distributed extraosseous data. Any changes in NPV from immediately after treatment to day 30 were compared using paired *t*-tests after log-transformation of these non-normally distributed data. The log-transformed post-treatment NPV data were also compared with log-transformed intraprocedural V_240EM_ data using Pearson’s correlations. Any differences in imaging features between intra- and extraosseous responders and nonresponders were described qualitatively because the sample sizes of these subgroups were too small to justify the use of statistical tests.

## Results

### Patients and Treatments

**Figure 2:**
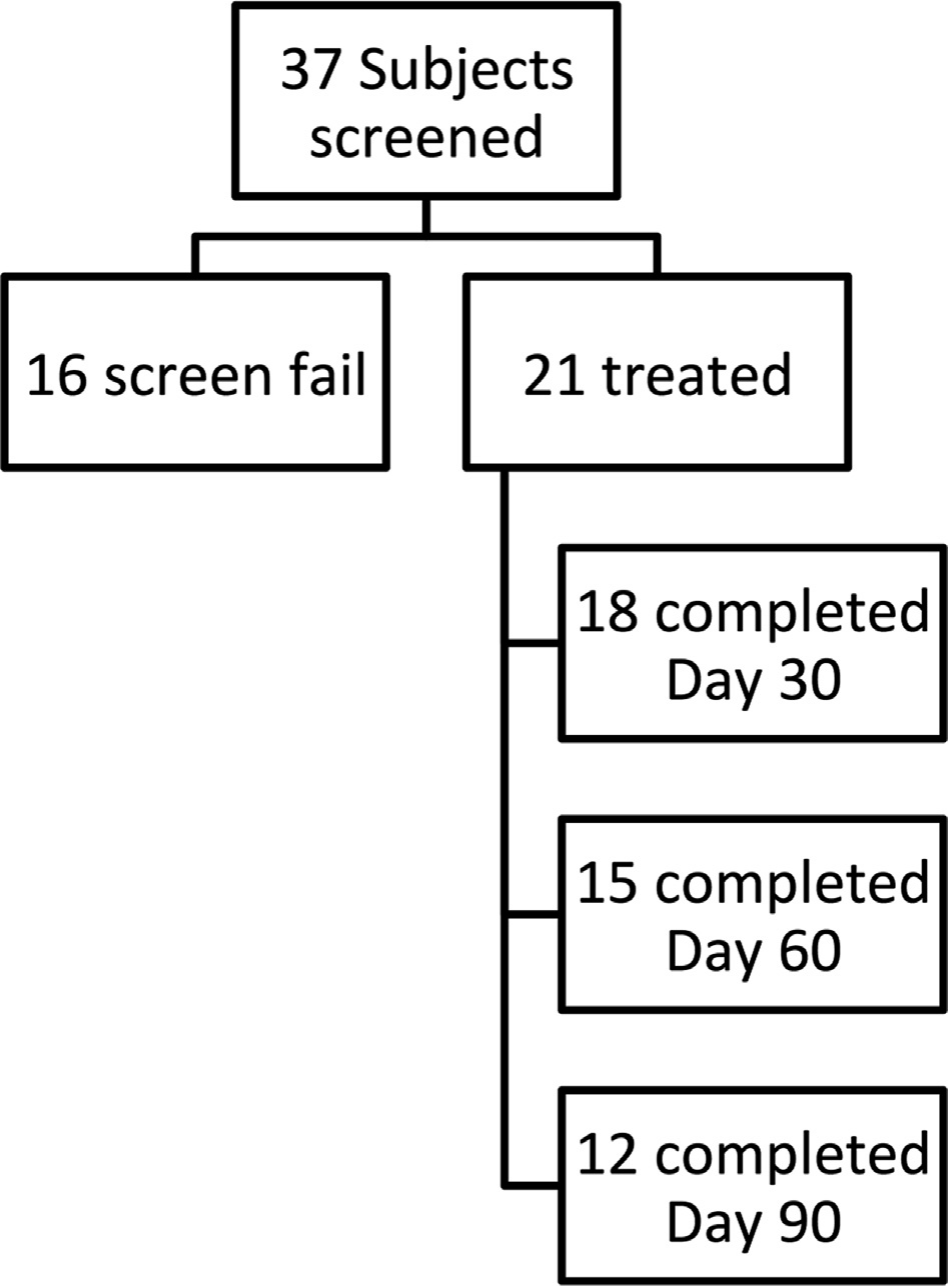
The schematic shows numbers of patients initially enrolled in the study and who subsequently progressed to treatment and attended for follow-up. Of the 9 patients who failed to complete day-90 follow-up, 5 were withdrawn from the study due to adverse events unrelated to treatment, 3 were referred to other interventions (radiation therapy), and 2 chose to withdraw from the study due to declining health.

Figure 2 shows the number of treated patients who completed follow-up. Table 3 gives differences in delivered treatments and PRFS-measured thermal parameters between patients with intraosseous (n = 9) and those with extraosseous (n = 12) tumors. Although treatments appeared more extensive in the extraosseous group, differences between groups were only significant in the number of delivered sonications and the measured thermal dose volumes (V_240EM_).

**Table 3:** Differences between Group Treatment Parameters and Tumor Diameters

	Intraosseous Group	Extraosseous Group	Differences
Delivered treatment parameters			
Depth of target, mm	35.9 ± 17.1	36.9 ± 16.3	*P* = 0.9
n sonications	15 ± 5	29 ± 15	***P* = 0.022**
Use of 12 mm diameter cells	1 *P*atient	4 *P*atients	
Treatment volume, mL	12.1 ± 13.3	16.4 ± 12.0	*P* = 0.21
Treatment time, min	70.6 ± 28.2	89.4 ± 42.0	*P* = 0.25
Mean power per sonication, W	69.8 ± 29.9	85.2 ± 46.8	*P* = 0.41
Total energy of treatment, kJ	23.5 ± 16.8	51.9 ± 49.3	*P* = 0.17
Measured thermal parameters			
V_240EM_, mL	5.4 ± 9.9 (range: 0.3-31.2)	13.9 ± 19.1 (range: 2.4-62.7)	***P* = 0.039**
T_M_, ^o^C	62.2 ± 5.9	60.6 ± 4.7	*P* = 0.51
Tumor diameter			
At baseline, mm	38.9 ± 12.8	55.7 ± 14.9	***P* = 0.012**
Significance of change in lesion diameter from baseline∗			
At day 30	*P* = 0.83	*P* = 0.13	
At day 60	*P* = 0.96	*P* = 0.47	
At day 90	*P* = 0.34	*P* = 0.63	

Note–Differences in characteristics and treatment parameters are shown for patients with intra- versus extraosseous tumors. Baseline tumor diameters and the lack of significant changes after treatment are also indicated. Bold indicates statistical significance.

T_M_ = mean maximum temperature from all sonication for each patient; V_240EM_ = thermal dose volume, estimated by calculating the product of 3 orthogonal maximum dimensions of the 240 equivalent minutes (EM) at 43^o^C thermal dose contour for each sonication, and cumlating the total value for all sonications for each patient.

∗ *P* values uncorrected for multiple time point comparisons. Unless otherwise specified, values shown are mean ± SD.

### Imaging Changes after Treatment

For both intra- and extraosseous tumors, mean maximum diameters measured using unenhanced T1W imaging were stable after treatment, with no significant differences from baseline at any post-treatment time-point (Table 3).

**Figure 3:**
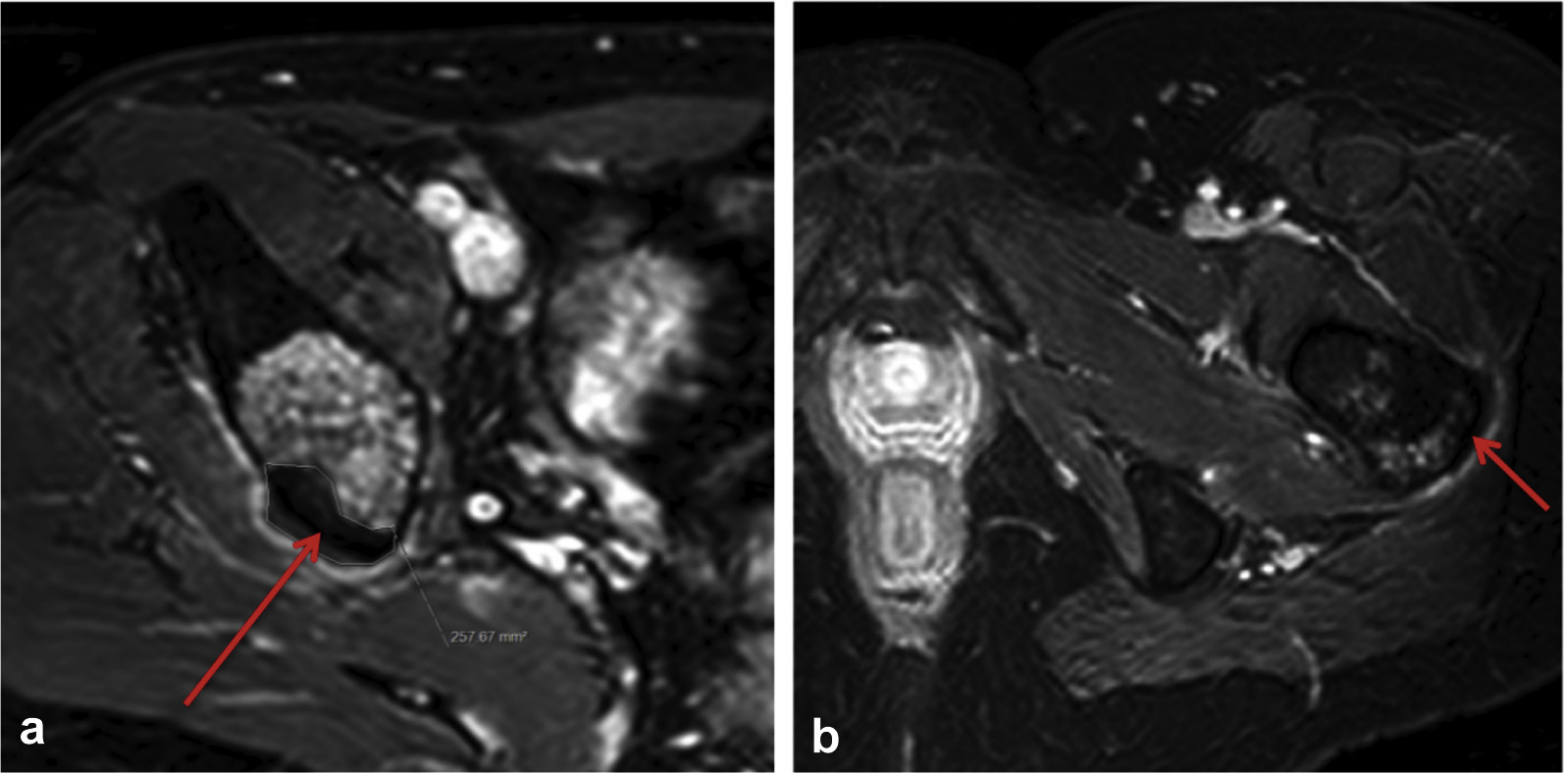
Example of a gadolinium-enhanced T1-weighted (Gd-T1W) image at 30 days after treatment. A clear focal region of nonenhancement (red arrows) was seen on either side of the bony cortex for intraosseous tumors (a) in a 36-year-old male with metastatic lung cancer, and (b) in a 45-year-old female with metastatic breast cancer. In both cases, a thin rim of enhancing tissue is also seen at the proximal border of the region of nonenhancement.

*Intraosseous Group****.—*** A nonperfused volume was recognized immediately after treatment on Gd-T1W images in 8 of 9 patients with intraosseous tumors. In 5 of 9 patients (56%), this was seen as a rind of nonenhancing tissue on either side of the osseous cortex, with a surrounding rim of enhancement at the proximal border of the unenhanced rind. In 3 tumors, ill-defined regions of nonperfusion were seen, and no contrast enhancement was evident in 1 tumor. By day 30 after treatment, a clear focal region of nonenhancement was present in 7 of 9 patients (78%) that persisted to days 60 and 90 in those with follow-up (Fig 3).

NPV measured immediately after treatment in 7 of 9 patients (mean 5.5 ± 9.9 mL; range: 0.1–27.3 mL) showed a strong and significant correlation with V_240EM_ measured during treatments (r = 0.87; *P* = 0.011). The NPV did not change significantly from immediately post-treatment to day 30, (mean 5.7 ± 8.8 mL; range: 1.0–25.3 mL; *P* = 0.25).

*Extraosseous Group***.—**All 12 extraosseous tumors were heterogeneous, with patchy regions of contrast enhancement and nonperfused regions of presumed necrosis on images acquired prior to the treatment day. These tumors showed no visibly identifiable changes on post-treatment scans in 7 of 12 patients (58%); in 2 patients, there was a grade-1 change, and in 3 patients there was grade-2 change. By day 30 after treatment, there was some evolution of image appearances in the 9 patients with imaging data: in 2, images still showed no change from baseline, in 4, there were grade-1 image changes, in 2, there were grade-2 image changes, and in 1, images showed the re-establishment of pretreatment enhancement after grade-1 change had been seen immediately post-treatment.

### Response to Treatment

**Figure 4:**
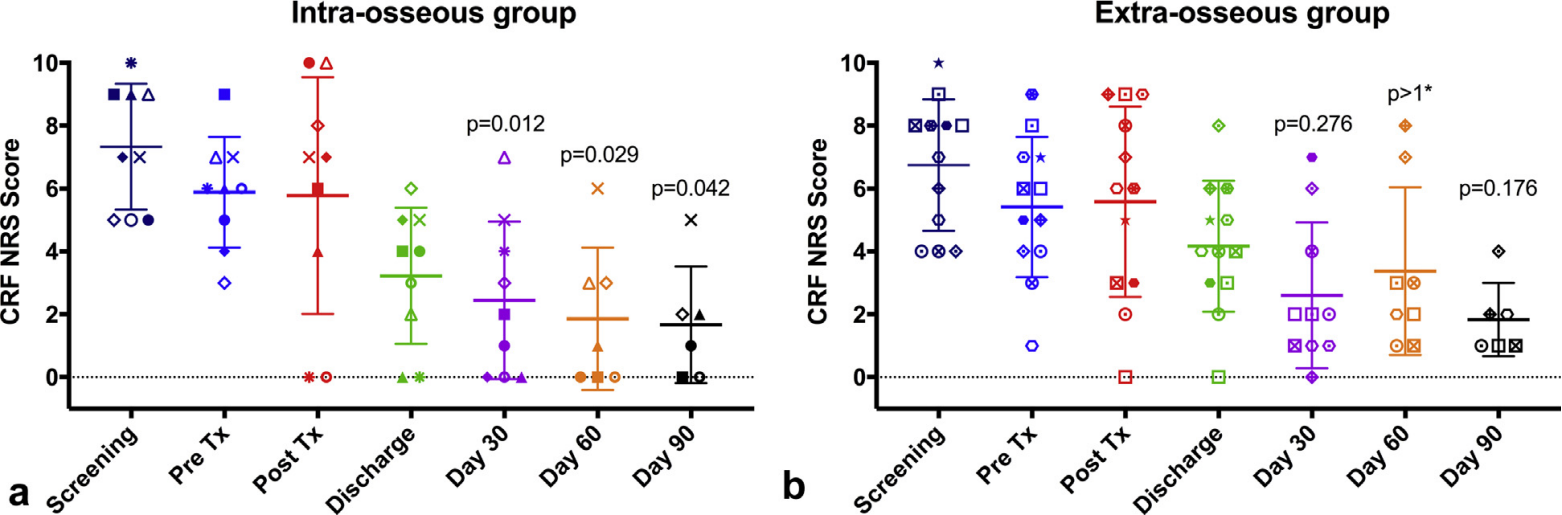
Pain scores recorded in the case report form (CRF) for the treated tumor for **(a)** 9 patients in the intraosseous group and **(b)** 12 patients in the extraosseous group. In each diagram, the horizontal lines show the mean ± SD scores, and a discrete marker shape is used to show the individual score for each patient at each time point. At days 30, 60, and 90 after treatment, scores were significantly lower than those in pretreatment (Pre Tx) for the intraosseous group but not for the extraosseous group. The *P* values have been corrected for multiple comparisons: *P* >1* indicates that the uncorrected *P* value was already >0.34. NRS = numerical rating scale.

**Figure 5:**
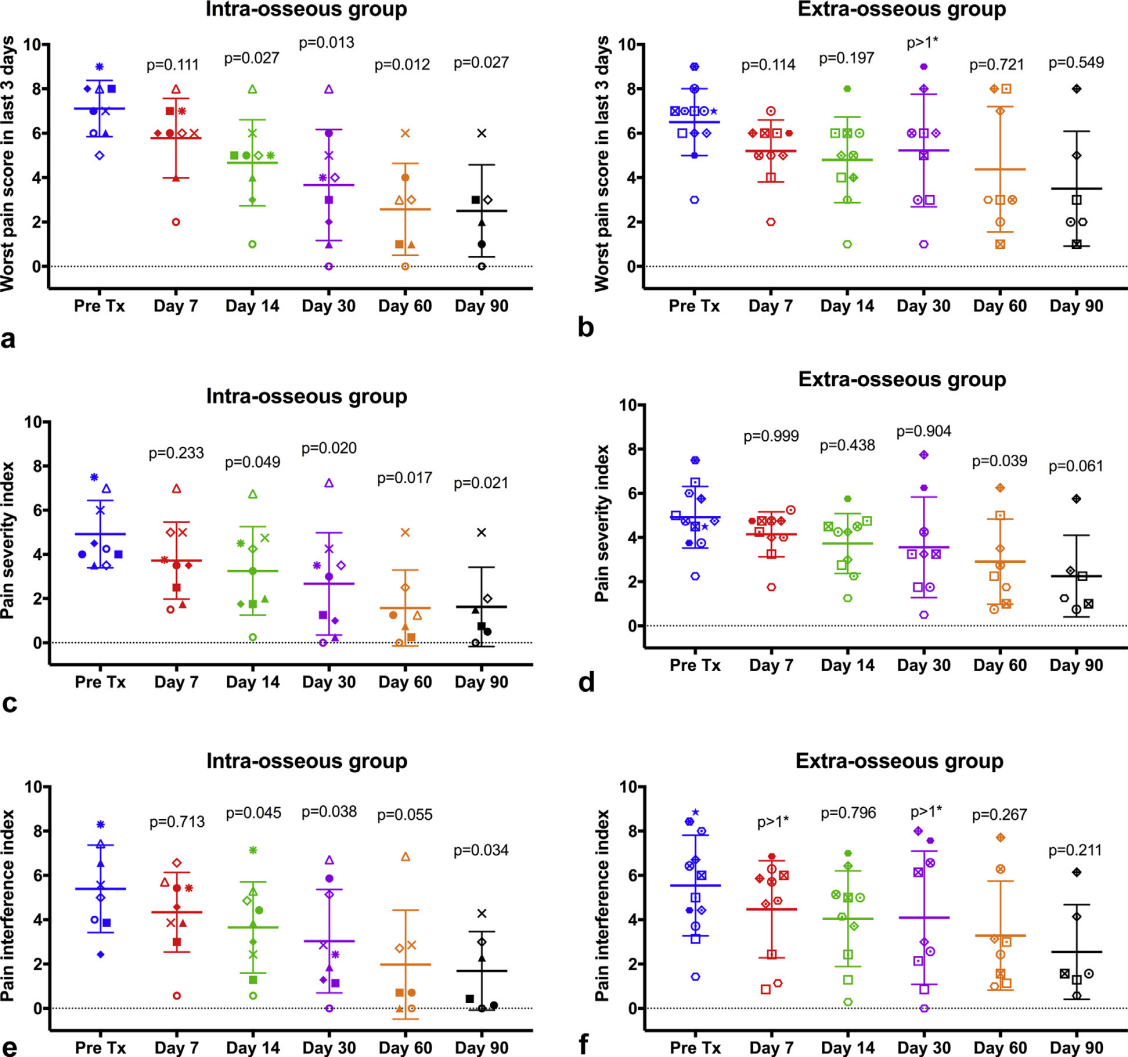
BPI-SF pain metrics for **(a,c,e)** intraosseous and **(b,d,f)** extraosseous groups. Worst pain, pain severity index, and pain interference indexes were all significantly improved by day 14 after treatment for the intraosseous group but not for the extraosseous group. Horizontal lines show the mean ± SD scores, and the discrete marker shapes show individual scores for each patient. The *P* values have been corrected for multiple comparisons: *P* >1* indicates that the uncorrected *P* value was >0.20. BPI-SF = Brief Pain Inventory short form.

**Figure 6:**
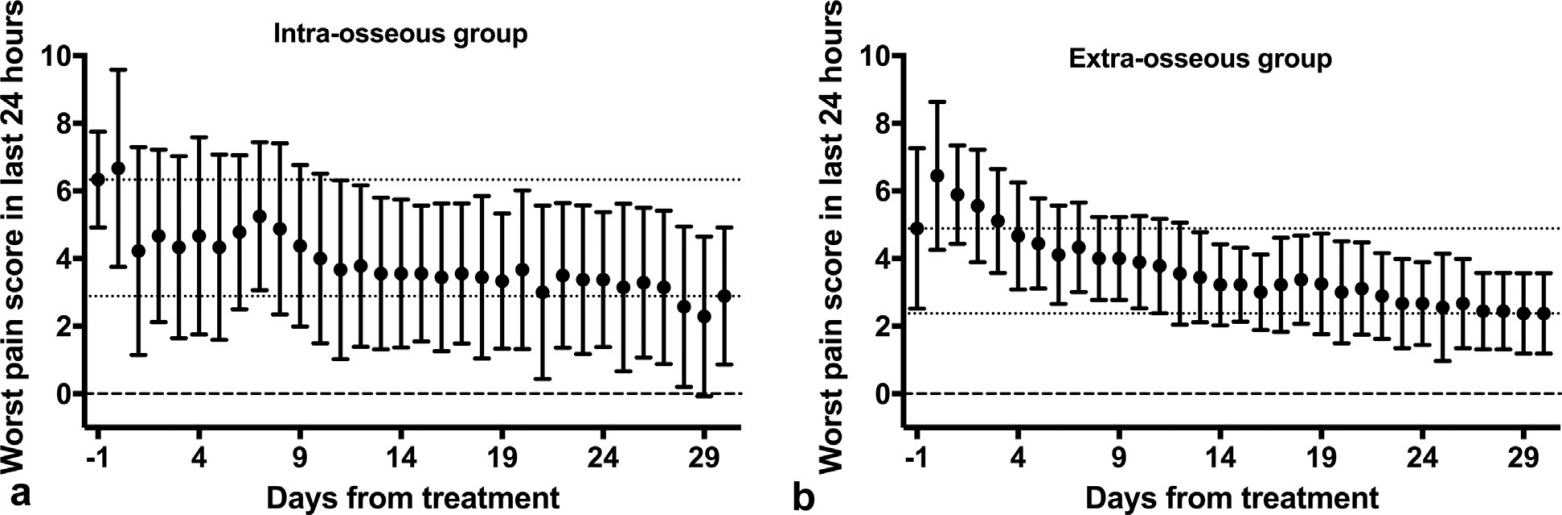
Mean ± SD 24-hour worst pain scores recorded in the patient diaries for **(a)** the intraosseous group and the **(b)** the extraosseous group. Day -1 shows the score recorded on the treatment day before treatment, whereas day 0 was the score recorded on the same day after treatment. The dotted lines show the mean pretreatment and day-30 scores. An improvement of >2 points in pretreatment scores was seen 1 day after treatment for the intraosseous group but not until day 22 for the extraosseous group. The pretreatment score for the extraosseous group appears artificially low in comparison with baseline CRF and BPI-SF scores. However, even if the post-treatment score had been used as the baseline, scores improved more gradually in this group, compared to the intraosseous patients. CRF = case report form; BPI-SF = Brief Pain Inventory short form.

Pain scores recorded on the CRFs in both groups showed reduction (Fig 4a,b), but differences from baseline at days 30, 60, and 90 were only significant for the intraosseous group (*P* = 0.012, *P* = 0.029, *P* = 0.042, respectively). The same pattern was seen for pain scores recorded in the BPI-SF, with significant reductions in worst pain, pain severity index, and pain interference index at every time point from day 14 after treatment only for the intraosseous group (Fig 5a–f). The daily worst pain scores recorded in the patient diaries also showed much earlier onset of pain relief in the intraosseous, as opposed to the extraosseous group, with an improvement of >2 points reported 1 and 22 days after treatment, respectively (Fig 6a,b).

**Figure 7:**
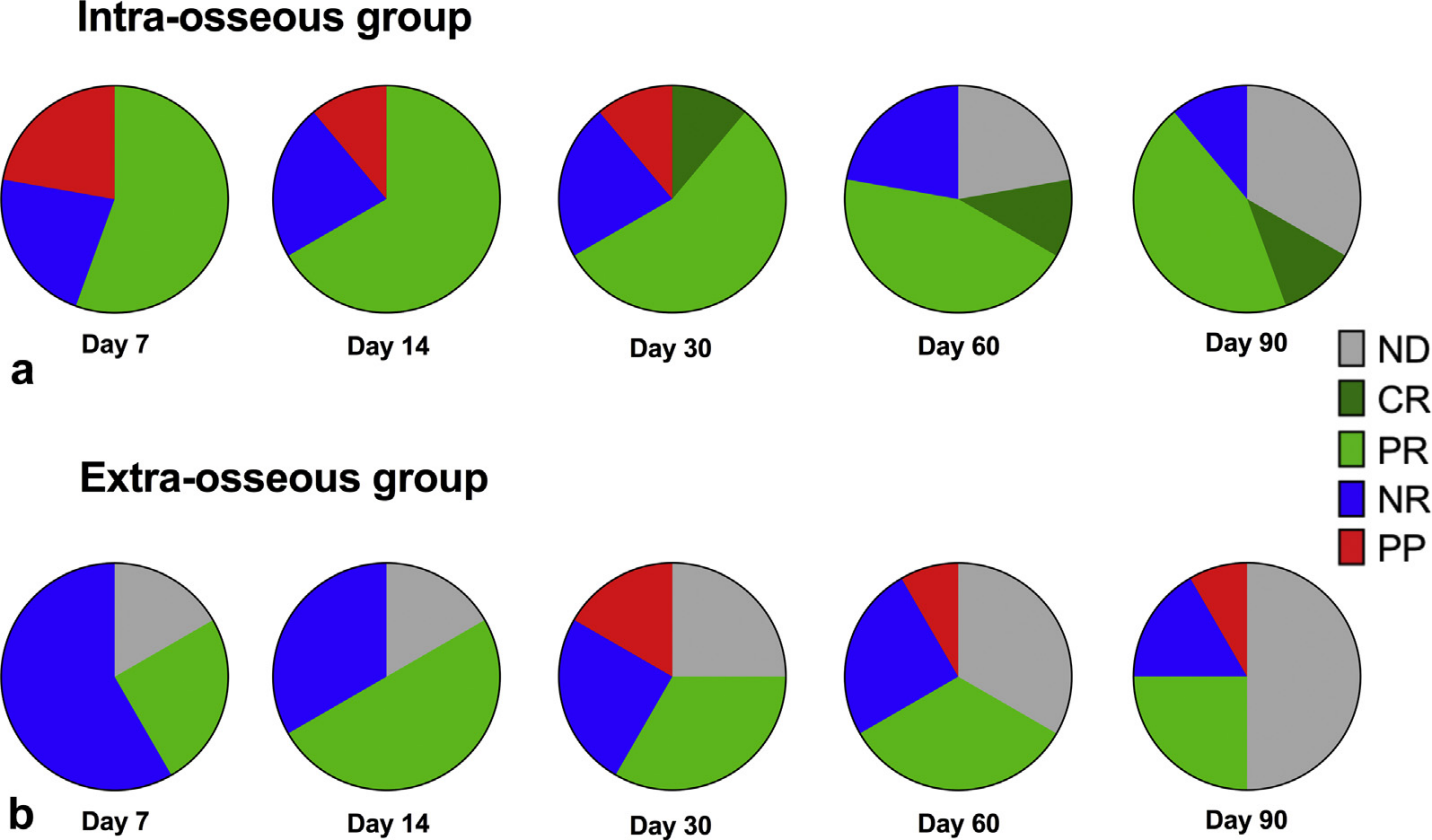
Treatment response classification for **(a)** intraosseous and **(b)** extraosseous groups. The proportion classified as responders at each time point is shown by the dark and light green segments, which indicate a complete response (CR) or a partial response (PR), respectively. The nonresponders (NR [no response]) are shown in blue and pain progression (PP) is shown in red. The gray segments show the patients who did not have follow-up data (ND [no data]) at each time point. These show higher response rates for the intraosseous group at every time point, even though 2 patients initially had a flare of increased pain (which subsequently resolved). More patients in the intraosseous group completed follow-up than in the extraosseous group (where 2 patients did not complete day 7, and 50% were withdrawn by day 90).

Six of nine intraosseous patients (67%) were classified as responders at day 30, compared to 4 of 12 patients (33%) in the extraosseous group. The latter patients also had a higher withdrawal rate from the study, with only 50% achieving day-90 follow-up, compared to 70% of intraosseous patients (Fig 7**)**. For both groups of patients, there were no clear differences in Gd-T1W imaging changes after treatment in those classified as responders or nonresponders at day 30.

### Adverse Events

There were no treatment-related serious AEs reported in the 21 patients. Of 5 AEs related to or possibly related to treatment in 4 patients, 4 were reports of pain after treatment in intraosseous patients and 1 of temporary numbness of the buttock after treatment of a sacral metastasis in an extraosseous patient. There were no fractures or skin burns after treatment, although day-30 imaging indicated possible thermal injury to adjacent subcutaneous fat tissues in 1 extraosseous patient. As expected in this population, the rate of AEs unrelated to treatment was higher (42 AEs in 14 patients, mainly relating to progression of underlying disease).

## Discussion

This study demonstrated significant differences in pain relief for patients with intra- versus extraosseous bone metastases treated palliatively with MRgHIFU. The improvements in pain scores for the treated tumors (measured from the CRF) and in global pain (measured from the BPI-SFs) seen in both groups showed significant changes from baseline at days 14, 30, 60, and 90 only in patients with intraosseous tumors. This was reflected in patients’ diaries, which showed that changes occurred much earlier and with clinically relevant and important improvements 23, 24 being seen within 1 day of treatment. The rapid onset of improvement in patients with painful intraosseous tumors constitutes a major advantage of the HIFU technique but appears harder to achieve in patients with extraosseous tumors.

The response rate of 67% for the intraosseous patients at day 30 is comparable with rates from other studies that have included heterogeneous populations 4, 5, 6, 7, 9, 25. Furthermore, of the 3 intraosseous nonresponders at day 30, 1 had achieved response by day 60 (sustained at day 90), whereas the 2 remaining patients had each reported 5- and 3-point reductions in focal pain at the treated site at day 60, but both also required >25% increase in analgesia for worsening pain in other regions and were therefore classified as nonresponders.

The lower response rate of 33% for the extraosseous patients at day 30 did not improve at later follow-up. Of the 3 patients classified as nonresponders at day 90, 2 experienced a reduction in pain score of only 1 point, without a change in analgesia, whereas 1 patient had both increased pain scores and increased analgesia, making all 3 true nonresponders. The high rate of withdrawal from the study (50% by day 90) because of disease progression is testament to the fact that these were patients with end-stage disease. Consequently, there were numerous AEs unrelated to treatment reported in this group.

If HIFU thermally denervates the periosteum 2, 26, it is unsurprising that better response rates were seen in the intraosseous group. Furthermore, as the treatments delivered to patients with the larger extraosseous tumors were not significantly more extensive than those delivered to patients with the smaller intraosseous tumors, an insufficient proportion of the soft tissue tumors may have been targeted to elicit a response, either from a debulking effect or from alterations in the release of proinflammatory signaling molecules 26, 27. A larger relative extent of thermal dose volume may be needed to achieve pain control in these soft tissue tumors. Although more aggressive treatments are technically feasible, they also risk a greater rate of AEs. However, the aim of achieving local tumor control as well as pain palliation has already been highlighted as a research priority for MRgHIFU therapy for painful bone metastases (1).

In the intraosseous patients, thermal neurolysis was probably achieved, given that clear regions of focal nonenhancement (NPV) were seen immediately after treatment in 5 of 9 patients and by day 30 in 7 of 9 cases. The NPV was significantly correlated with thermal dose volumes (V_240EM_) but did not translate to an indication of treatment response. The small sample size or the potential confounding effects of response classification may explain this. Alternatively, it may be that periosteal ablation is achieved regardless of visible soft tissue damage. This reinforces observations in previous studies in which NPV was unrelated to pain score (15) and did not differ between responders and nonresponders (8).

These results suggest that post-treatment imaging, even using contrast-enhanced techniques, may not be informative about treatment efficacy for pain palliation of bone metastases. Also, it can play only a limited role in ensuring patient safety, given that the opportunity to modify treatments has passed. It may serve a purpose in early recognition of complications (e.g., a large immediate post-treatment NPV involving adjacent muscle in 1 intraosseous patient could have prompted proactive, early referral for physiotherapy to reduce muscle stiffness). However, this could also have been recognized during treatment because of the high intraprocedural V_240EM_. Thus, PRFS data may potentially help flag the likelihood of collateral tissue damage at a time when treatments could still be modified or curtailed. Removing the requirement for post-treatment imaging assessments would reduce the burden of imaging appointments on patients and spare them from repeated administrations of contrast agents at a time when use of these agents is increasingly scrutinized. It would also reduce the resource requirement for institutions delivering these treatments.

The main limitation of this study was the small sample size in each group, caused by difficulties in recruiting sufficient suitable patients within a reasonable time scale and high rates of study withdrawal. A potential source of bias in the findings was that the patients in each group were not matched and that the extraosseous patients might have had more advanced disease. Response relative to ablative thermal dose per tumor volume and length of destroyed cortex needs to be established in these patients. Also, there was no mechanism for separating use of analgesics for pain in a target tumor from pain in nontarget regions, potentially confounding response assessment in some cases. In addition, the estimated V_240EM_ was only an approximation of thermal dose volume, chosen because it could be quickly and easily obtained on the Sonalleve console as treatments progressed. More accurate and robust methods for calculating thermal dose volume after treatment completion already exist and could potentially be made available in a more timely fashion.

This study documents differences between MRgHIFU treatments delivered to patients with intraosseous bone metastases and to those with extraosseous bone metastases. Response rates for patients with intraosseous tumors were considerably better than for those with extraosseous ones, who may require more aggressive treatments to achieve pain control. Imaging changes differed between the groups but did not indicate treatment response. Follow-up scanning after treatment may therefore be required only for assessing disease progression or adverse events, rather than for monitoring on-going treatment efficacy.

## Figures and Tables

**Table E1:** Summary of Acquired Sequence Parameters at 1 Site

Parameter	T1W	PRFS	Gd-T1W
TR, ms	3.5	25	5.4
TE, ms	2.3	16	2.6
FA, ^o^	7	18	12
Fat suppression	-	ProSet	SPAIR
Frequency offset, Hz	-	-	220
EPI/TFE factor	-	11	18
Voxel size, mm^3^	1.25 × 1.75 × 1.25	2.1 × 2.1 × 7.0	1.5 × 1.5 × 3.0
FOV, mm	240 × 320 × 140	400 × 300 × 7 each stack	220 × 220 × 105
NSA	2	2	1
Number of slices	112	4	70
Scan duration, min:s	2:30–3:10∗	0:03†	1:55

Note–Summary of acquired sequence parameters at 1 site using a 3-T Achieva system (Philips Healthcare, Best, the Netherlands). Comparable sequence parameters wereimplemented at the other 2 sites, 1 of which used a 3-T system and the other used a 1.5-T system.

EPI/TFE = echo planar imaging/turbo field echo; FA = flip angle; FOV = field of view; Gd-T1W = gadolinium-enhanced T1-weighted; NSA = number signal averages; PRFS = proton resonance frequency shift; ProSet = principle of selective excitation technique; SPAIR = spectral attenuated inversion recovery; TE = echo time; TR = repetition time.

∗ Scan duration was influenced by the amount of oversampling required to avoid wrap artifacts,

† Images were updated every 3 s (dynamic scan time); total acquisition time was determined by the number of frames of imaging (dynamics) acquired.
